# Performance on the DANA Brief Cognitive Test Correlates With MACE Cognitive Score and May Be a New Tool to Diagnose Concussion

**DOI:** 10.3389/fneur.2020.00839

**Published:** 2020-09-02

**Authors:** Jennifer R. Pryweller, Brandon C. Baughman, Samuel D. Frasier, Ellen C. O'Conor, Abhi Pandhi, Jiajing Wang, Aimee A. Morrison, Jack W. Tsao

**Affiliations:** ^1^Department of Neurology, College of Medicine, University of Tennessee Health Science Center, Memphis, TN, United States; ^2^Semmes Murphey Clinic, Memphis, TN, United States; ^3^Department of Otolaryngology – Head and Neck Surgery, Naval Medical Center Portsmouth, Portsmouth, VA, United States; ^4^Division of Biostatistics, Department of Preventative Medicine, University of Tennessee Health Science Center, Memphis, TN, United States; ^5^Department of Obstetrics and Gynecology, Hospital of the University of Pennsylvania, Philadelphia, PA, United States; ^6^Memphis Veterans Affairs Medical Center, Memphis, TN, United States; ^7^Children's Foundation Research Institute, Le Bonheur Children's Hospital, Memphis, TN, United States

**Keywords:** mTBI, concussion, DANA, MACE, military, neurocognitive assessment

## Abstract

Nearly 380,000 U.S. service members between 2000 and 2017 were, and at least 300,000 athletes annually are, diagnosed with concussion. It is imperative to establish a gold-standard diagnostic test to quickly and accurately diagnose concussion. In this non-randomized, prospective study, we examined the reliability and validity of a novel neurocognitive assessment tool, the Defense Automated Neurobehavioral Assessment (DANA), designed to be a more sensitive, yet efficient, measure of concussion symptomatology. In this study, the DANA Brief version was compared to an established measure of concussion screening, the Military Acute Concussion Evaluation (MACE), in a group of non-concussed service members. DANA Brief subtests demonstrated low to moderate reliability, as measured by intra-class correlation coefficient (ICC; values range: 0.28–0.58), which is comparable to other computerized neurocognitive tests that are widely-implemented to diagnose concussion. Statistically significant associations were found between learning and memory components of the DANA Brief and the diagnostic MACE cognitive test score (DANA Brief subtests: CDD: *R*^2^ = 0.05, *p* = 0.023; CDS: *R*^2^ = 0.10, *p* = 0.010). However, a more robust relationship was found between DANA Brief components involving attention and working memory, including immediate memory, and the MACE cognitive test score (DANA Brief subtests: GNG: *R*^2^ = 0.08, *p* = 0.003; PRO: *R*^2^ = 0.08, *p* = 0.002). These results provide evidence that the DANA Rapid version, a 5-min assessment self-administered on a hand-held portable device, based on the DANA Brief version, may serve as a clinically useful and improved neurocognitive concussion screen to minimize the time between injury and diagnosis in settings where professional medical evaluation may be unavailable or delayed. The DANA's portability, durability, shorter test time and lack of need for a medical professional to diagnose concussion overcome these critical limitations of the MACE.

## Introduction

According to the Defense and Veterans Brain Injury Center, US service members (a person serving in the armed forces) sustained nearly 380,000 cases of traumatic brain injury (TBI) between 2000 and 2017. Of these, 82.3% were classified as mild (1). According to the Department of Defense guidelines, mild traumatic brain injury (mTBI), also called concussion, is diagnosed when an injury event has occurred and the individual experiences one or more of the following: (1) an alteration of consciousness lasting <24 h, (2) loss of consciousness (LOC) from 0 to 30 min, or (3) post traumatic amnesia from 0 to 1 days (2).

Concussion is also common among athletes. According to the Centers for Disease Control and Prevention (CDC), at least 300,000 athletes are diagnosed with concussions per year (3). Sport-related concussions are defined as a mTBI induced by biomechanical forces and, because of their potential to have rapidly changing and sometimes unpredictable clinical symptoms, are often difficult to diagnose (4, 5). Exposure to these biomechanical forces triggers a complex neurometabolic cascade, described by Giza and Hovda as a series of microcellular events, including ionic shifts, abnormal energy metabolism, diminished cerebral blood flow, and impaired neurotransmission (6). Because of the underlying pathophysiology of mTBI, conventional imaging (i.e., CT, MRI) is not considered diagnostic and frequently does not provide useful clinical information. Additionally, reliance on athlete/patient self-report is itself problematic. Delaney et al. report that 63–70% of contact sport athletes reported symptoms; however, only 20–23% realized that these symptoms constituted concussion (7). In some cases, concussion symptoms are known but not reported for various reasons. McCrea et al. reported on confidential surveys of 1,500 high school football players, 40% of which acknowledged the presence of concussive symptoms but deliberately failed to disclose the information out of fear of being removed from play (8).

A concussion can result in a variety of acute symptoms, including, but not limited to, nausea, headache, dizziness, fatigue, balance problems, sensitivity to light, and memory and concentration deficits (9, 10). Typically, symptom recovery occurs within 7–10 days post-injury (11, 12). Recovery of cognitive function, however, can take anywhere from 1 to 3 months (13, 14). It is, therefore, possible that individuals may present as clinically recovered (asymptomatic), but still have lingering cognitive deficits. In the absence of a gold-standard biomarker for concussion, clinicians often utilize neurocognitive testing methods to diagnose and determine recovery status (4). The utility of objective neurocognitive testing is well-established, with several studies showing measurable decrements in performance during the initial hours to weeks post-injury (15–17). Additionally, resolution of measured cognitive impairment via objective assessment tends to be on the magnitude of 2–14 days, which is similar to symptom recovery (11).

The United States Department of Defense currently uses the Military Acute Concussion Evaluation (MACE) as its designated method for evaluating suspected concussion (18–20). The MACE is composed of three parts: history of the injury, cognitive examination, and neurological screening. The cognitive component of the MACE includes questions that are heavily weighted toward memory functions. The aggregate of these subtests generates the MACE cognitive test score, which can help to determine cognitive impairment. There are normed cut-off values for the MACE which, when abnormal, are helpful in clinical evaluation. Normal values, however, cannot rule out the presence of concussion, which remains a clinical diagnosis based upon the history of events surrounding the injury. Also, if the MACE is administered more than 12 h after a concussive event, the cognitive examination section has been shown to lack sensitivity and specificity, thereby limiting its utility for early and accurate identification of concussion (20, 21). Additionally, the MACE must be administered by a trained professional, decreasing its functionality as a quick and easy method to diagnose concussion and track its recovery.

Unlike the MACE, the Defense Automated Neurobehavioral Assessment (DANA) is a durable and portable neurocognitive assessment tool that can be self-administered on a handheld device, making it well-suited for military operations and/or extreme environments as well as the sideline of a sports field. The DANA has three test battery versions: the DANA Rapid (5 min), DANA Brief (15 min), and DANA Standard (45 min). Each test varies in subtest composition and duration. The DANA Rapid focuses on basic reaction time measures (one of the more sensitive measures of impairment after concussion) and is the shortest of the three batteries (5 min). The DANA Brief builds on the Rapid and consists of a 15-min battery of tests plus additional psychological screening tools for post-traumatic stress disorder (PTSD), depression, and insomnia. The DANA Standard is the most comprehensive of the versions, containing the DANA Standard subtests as well as additional neurocognitive and psychological tests, and takes approximately 45 min to administer (22).

Since it is well established that cognitive impairment as a result of concussion is quite heterogeneous, previous studies have suggested concussive cognitive sequelae tend to follow a subcortical phenotype characterized by difficulties in attention, processing speed, executive function and memory recall (vs. encoding). While the MACE assesses attention, learning and memory items, the DANA extends the breadth of cognitive task domains that are assessed, with the inclusion of measures of reaction time, visuomotor processing speed, and measures of executive function (i.e., cognitive control). A more detailed discussion of the individual components in the MACE and DANA is presented in the Methods section below.

Because of the clinical ramifications of undiagnosed concussion, which can lead to further brain injury, it is imperative to establish a gold-standard diagnostic test to quickly and accurately diagnose this injury. A gold-standard neurocognitive test should ideally rely on purely objective measures and be rapidly self-administrable in diverse environments where professional medical evaluation is unavailable or delayed. This would benefit service members, professional athletes, and other individuals who have sustained a concussion by minimizing the time between injury and diagnosis.

This study was a prospective, non-randomized study comparing results from the DANA Brief subtests to the MACE cognitive test score in a group of non-concussed military service members in a remote, deployed environment. The primary objective of this study was to determine which individual of DANA Brief subtests were most correlated with the MACE cognitive test score in a remote, isolated, austere environment. We hypothesized that subtests related to memory on the DANA Brief [Code Substitution Simultaneous (CDS) and Code Substitution Delayed (CDD)] would be significantly correlated with the MACE cognitive test score. This hypothesis was based on the fact that the CDS and CDD assess short-term memory, where roughly 57% of the MACE cognitive test score is memory-task dependent. The investigators acknowledged that the cognitive constructs assessed by the CDS and CDD are diverse. Specifically, the CDS involves visual processing speed and attention. Further, the memory paradigm assessed in the CDS and CDD is primarily one of incidental learning and memory, as opposed to the more intentional learning that is screened as part of the MACE cognitive subtests. Nonetheless, we hypothesized that there would be greater overlap between the CDS and CDD DANA Brief subtest scores and the MACE cognitive test score, than between the DANA Brief simple reaction time subtests [Simple Reaction Time (SRT1 and SRT2)] and the MACE cognitive test score. A secondary objective of this study was to examine successive iterations of performance on the DANA Brief in the same group of volunteers. We also hypothesized that repeated administrations of the DANA Brief would result in improvements in an individual's performance due to a learning curve.

## Materials and Methods

### Study Volunteers

This study was approved by the Institutional Review Board of the United States Army Medical and Materiel Command, which had regulatory oversight over all human subjects research in Afghanistan. A total of forty male United States Marines from an infantry unit deployed to Afghanistan volunteered to participate in the study (mean age ± SD = 22.6 ± 3.8). Inclusion criteria were defined as active duty military service members who had a Glasgow Coma Scale score of at least 15 at the time of consent and who had not experienced blast exposure, collisions, rollovers or direct blows to the head within the past 24 h (2, 22). Exclusion criteria included sustaining a concussion within 3 months prior to study participation.

### Military Acute Concussion Evaluation (MACE)

The MACE is a three-part, 15-min test battery based on the SAC, and is the primary screening tool used in military populations for the acute evaluation of concussion. The first part consists of open-ended and yes or no clinical history questions, such as how the head injury occurred, what symptoms were experienced, and whether there was a LOC. The second part is a scored cognitive exam with four subtests: orientation (5 points); immediate verbal memory (15 points; recall of a 5-item word list presented over three consecutive learning trials); concentration (5 points; reverse digit sequencing and reversal naming of calendar months); and delayed memory (5 points; recall of the previous 5-item word list). From this exam, a total score out of 30 points, called the MACE cognitive test score, is generated based on adding the scores from each subtest. The mean MACE cognitive test score in military populations is 28, and a score of <25 points (2 standard deviations from the mean) represents potentially clinically relevant cognitive impairment. A neurologic exam follows the cognitive exam with special focus on examining pupil reactivity, speech fluency, and motor deficits (18).

### Defense Automated Neurobehavioral Assessment (DANA) Brief

The DANA Brief consists of seven subtests, including four from the DANA Rapid: Simple Reaction Time (SRT) assessing basic reaction time (administered twice, once at the beginning of the test battery and once at the end, giving two subtests, SRT1 and SRT2), Procedural Reaction Time (PRO) measuring attention and processing speed, and Go/No-Go (GNG) which assesses speed, accuracy, and response omissions and commissions. Additional subtests on the DANA Brief include Code Substitution Simultaneous (CDS) assessing visual scanning, processing speed, attention, learning and immediate memory; Spatial Discrimination (SPD) which measures spatial manipulation, and Code Substitution Delayed (CDD) which assesses visual recognition memory. It also includes a Patient Health Questionnaire (PHQ), a Primary Care PTSD screen (PC-PTSD), and an Insomnia Screening Index (ISI).

Three scores are calculated and reported for each DANA subtest: mean reaction time (RT; ms), RT correct (RT of correct responses; ms), and a mean throughput score, which is calculated using the following equation:

(1) $$\begin{array}{r} Mean\;throughput\;score\;=\;\frac{\%\;correct\;responses}{mean\;RT\;correct} \end{array}$$

### Test Administration

The MACE was administered to forty non-concussed service member volunteers by a clinician in a remote, deployed environment medical tent, which was the standard medical treatment (Role 1) field facility. Immediately following the MACE administration, the DANA Brief was self-administered on a commercially available handheld computer with a touch screen (Trimble Nomad). The total administration time for both the MACE and DANA Brief was 30 min. All forty volunteers were re-assessed on a single follow up day, 24 h after baseline assessment, for the second iteration of testing. This testing session consisted of a repeat administration of the MACE and DANA Brief. Twenty of the forty volunteers were assessed again on a subsequent follow-up day, 48 h after baseline assessment, for a total of three MACE and DANA Brief test iterations. Testing order was not randomized (the MACE was always administered prior to the DANA Brief).

### Statistical Analysis

For each DANA Brief subtest, a repeated measures ANOVA was used to assess the association between each DANA Brief subtest mean throughput score (subtest_score) and volunteer's corresponding MACE cognitive test score (MCTS). The full model of each DANA brief subtest included the following dependent and independent variables: DANA Brief subtest mean throughput score (continuous variable), iteration (categorical variable), age (continuous variable) and the interaction term between DANA Brief subtest score and iteration (Equation 2).

(2) $$\begin{array}{r} MCTS=\beta_{0}+\beta_{1}subtest\text{\_}score+\beta_{2}iteration\\ +\left(\beta_{3}subtest\text{\_}score*iteration\right)+\;\beta_{4}age+\beta \; \end{array}$$

For each DANA Brief subtest, the final mixed model was determined by a backward model selection and included the following dependent and independent variables: MCTS (continuous variable), DANA Brief subtest mean throughput score (continuous variable) and age (continuous variable) (Equation 3). Reliability of the final mixed model was evaluated based on the intraclass correlation coefficient (ICC) in the final mixed model with 95% confidence intervals calculated based on a single rater, absolute agreement, one-way random effects model. Validity was assessed based on the correlation between each of the DANA Brief subtests and the MACE cognitive test score. Eta-squared was calculated to obtain effect size, which measures the strength of the relationship between DANA Brief subtests and the MACE cognitive test score. Statistical analyses were performed using SAS 9.4.

(3) $$\begin{array}{r} {MCTS=\;\beta }_{0}+\beta_{1}subtest\text{\_}score+\;\beta_{2}age+\;\beta \end{array}$$

### Results

Group summary statistics for DANA Brief mean throughput scores and the MACE cognitive test scores for each test iteration are reported in Table 1. DANA Brief performance was not significantly different between baseline and testing 24 to 48-h later (Table 2). DANA Brief subtests demonstrated low to moderate reliability, with a range of ICCs from 0.28 to 0.58 (Table 1). The association between CDD (*R*^2^ = 0.05, *p* = 0.023), CDS (*R*^2^ = 0.10, *p* = 0.010), GNG (*R*^2^ = 0.08, *p* = 0.003), PRO (*R*^2^ = 0.08, *p* = 0.002) DANA Brief mean throughput scores and the corresponding MACE cognitive test score were all statistically significant; however, SPD, SRT1 and SRT2 mean throughput scores were not significantly associated with the MACE cognitive test score. Eta-squared values for the DANA Brief subtests ranged from 0.4 to 9.5%, where CDS (η^2^ = 9.5%) and GNG (η^2^ = 7.4%) had the highest effect sizes, explaining the proportion of total variance can be accounted for by DANA Brief subtests in the final mixed model. Results are reported in Table 3.

**Table 1:** Group summary statistics and reliability, as measured by intra-class correlation coefficient (ICC), for DANA Brief mean throughput scores and the MACE cognitive test scores for each test iteration.

***N***		**Test Iteration**		
	**1**	**2**	**3**	
	**40**	**40**	**20**	
**DANA Brief Subtest**	**Mean** **±** **SD**	**Mean** **±** **SD**	**Mean** **±** **SD**	**ICC (95% CI)**
CDD	54.76 ± 14.86	54.92 ± 13.68	41.94 ± 18.18	0.28 (0.09, 0.49)
CDS	49.42 ± 8.62	51.77 ± 7.79	48.39 ±8.45	0.58 (0.40, 0.73)
GNG	121.47 ± 15.72	121.78 ± 17.17	102.10 ± 20.46	0.58 (0.40, 0.73)
PRO	100.99 ± 15.18	104.52 ±13.37	98.84 ± 17.01	0.58 (0.40, 0.73)
SPD	35.65 ± 7.82	39.51 ± 9.20	37.06 ± 11.46	0.58 (0.40, 0.73)
SRT1	200.70 ± 24.03	194.82 ± 26.29	180.43 ± 30.20	0.58 (0.40, 0.73)
SRT2	192.56 ± 24.99	189.05 ± 28.24	156.28 ± 43.79	0.58 (0.40, 0.73)
**MACE Cognitive Test**	**Mean** **±** **SD**	**Mean** **±** **SD**	**Mean** **±** **SD**	
MCTS	27.10 ± 2.06	27.63 ± 1.73	26.90 ± 2.94	0.58 (0.40, 0.73)

*DANA Brief subtests: CDD, Code Substitution Delayed; CDS, Code Substitution Simultaneous; GNG, Go/No-Go; PRO, Procedural Reaction Time; SPD, Spatial Discrimination; SRT1, Simple Reaction Time (first administration); SRT2, Simple Reaction Time (second administration); MCTS, MACE cognitive test score*.

**Table 2:** Results of comparison between the DANA Brief subtest mean throughput scores and MACE cognitive test scores in the linear mixed model (Equation 2).

**Variables**	**β**	***p*-value**	**η^2^ (%)**	***R*^**2**^**
CDD	0.013	0.642	2.8	0.03
Iteration	−0.249	0.715	−1.7	
Iteration^*^CDD	0.009	0.480	−1.0	
Age	0.050	0.475	0.8	
CDS	0.085	0.047^*^	8.4	0.08
Iteration	0.865	0.435	−2.0	
Iteration^*^CDS	−0.015	0.493	−1.0	
Age	0.086	0.209	3.0	
GNG	0.054	0.017^*^	7.2	0.07
Iteration	1.831	0.125	−1.2	
Iteration^*^GNG	−0.013	0.210	−0.9	
Age	0.102	0.161	3.0	
PRO	0.073	0.002^*^	7.9	0.08
Iteration	2.218	0.066	−0.1	
Iteration^*^PRO	−0.021	0.078	1.0	
Age	0.076	0.249	3.0	
SRT1	0.024	0.096	2.3	0.03
Iteration	1.934	0.146	−0.5	
Iteration^*^SRT1	−0.009	0.208	1.4	
Age	0.052	0.456	1.1	
SRT2	0.018	0.250	4.0	0.04
Iteration	0.932	0.460	0.3	
Iteration^*^SRT2	−0.004	0.594	0.8	
Age	0.046	0.500	0.7	
SPD	0.082	0.068	0.5	0.01
Iteration	1.537	0.032^*^	−0.92	
Iteration^*^SPD	−0.037	0.053	0.1	
Age	0.069	0.351	2.5	

*β, validity; η^2^, effect size. DANA Brief subtests: CDD, Code Substitution Delayed; CDS, Code Substitution Simultaneous; GNG, Go/No-Go; PRO, Procedural Reaction Time; SPD, Spatial Discrimination; SRT1, Simple Reaction Time (first administration); SRT2, Simple Reaction Time (second administration)*;

* *p < 0.05*.

**Table 3:** Results of comparison between the DANA Brief subtest mean throughput scores and MACE cognitive test scores in the final linear mixed model (Equation 3).

**Variables**	**β**	***p*-value**	**η^2^ (%)**	***R*^**2**^**
CDD	0.027	0.023^*^	3.5	0.05
Age	0.051	0.461	0.9	
CDS	0.064	0.010^*^	9.5	0.10
Age	0.086	0.203	3.0	
GNG	0.030	0.003^*^	7.4	0.08
Age	0.097	0.179	2.9	
PRO	0.040	0.002^*^	6.9	0.08
Age	0.077	0.243	1.9	
SRT1	0.007	0.267	0.8	0.02
Age	0.053	0.449	0.6	
SRT2	0.010	0.071	2.7	0.04
Age	0.048	0.482	0.4	
SPD	0.017	0.463	0.4	0.02
Age	0.068	0.358	2.0	

*β, validity; η^2^, effect size. DANA Brief subtests: CDD, Code Substitution Delayed; CDS, Code Substitution Simultaneous; GNG, Go/No-Go; PRO, Procedural Reaction Time; SPD, Spatial Discrimination; SRT1, Simple Reaction Time (first administration); SRT2, Simple Reaction Time (second administration)*;

* *p < 0.05*.

## Discussion

While there is no gold-standard test for the assessment of concussion, any new tool has to be equivalent or better than established screening tools such as the MACE. This study examined the reliability and validity of a novel measure (the DANA Brief), designed to be a more sensitive, yet efficient, measure of cognitive performance following a concussion. In this non-randomized, prospective study, the DANA Brief was compared to an established measure of concussion screening (the MACE).

Low to moderate test-retest reliability of the DANA Brief subtests is an unsurprising reflection of ICCs commonly reported in studies of widely-implemented computerized neurocognitive tests (CNTs) to assess and diagnose concussion. The present study found ICC values that range from 0.30 to 0.63. Although results of repeat testing in non-concussed individuals should not change in theory, some inherent level of measurement error can be expected in any test and is reflected by low reliability (23). Reliability values associated with CNTs such as the DANA Brief may be biased by various factors. Given the conflicting implications of moderating variables in the present study, future studies would benefit from a larger sample size to increase power and allow for the comparison of these variables to better understand the basis of the DANA Brief subtest ICCs reported in this study.

In addressing the validity of the DANA Brief, of particular interest was the relationship between measures of incidental visual memory and recognition, inherent in several DANA Brief subtests and the MACE cognitive test. In this preliminary investigation, we found evidence to support our hypothesis regarding the correlation between learning and memory components of the DANA Brief (i.e., CDD, CDS) and the MACE cognitive test score. However, a more robust relationship was found between the GNG and PRO subtests and the MACE cognitive test score. This is not surprising given that several components of the MACE cognitive test score involve attention and working memory, including immediate memory. It is well established that attentional control and focus to task influences initial encoding and immediate memory performance. This is relevant in the current study, as 50% of MACE cognitive test score points come from immediate memory. Further, studies have suggested that the primary memory problem in concussed individuals tends to be one of initial encoding, requiring greater attentional demand, as opposed to delayed retrieval (24). Taken together, findings that DANA Brief measures of more direct reaction time and processing speed (SRT1 and SRT2) were not significantly associated with the MACE cognitive test score, and that GNG and PRO subtests explain the most variance in the final mixed model, suggests the DANA Brief is an instrument tapping an additional, unique cognitive construct relevant in the context of concussion/mTBI. These results, in the context of other study findings, further support preliminary evidence that the DANA Rapid version may serve as a clinically useful and improved neurocognitive concussion screen where professional medical evaluation is unavailable or delayed.

From a practical standpoint, the clinical definition of concussion has, historically, relied upon observable signs (e.g., LOC); however, we also know that a high percentage of concussions (90%) do not present with LOC, and the U.S. Department of Defense uses either an alteration of consciousness or LOC in its definition of concussion (11, 25, 26). Accordingly, there is a need for instruments that assess beyond observable symptoms. Based on our findings, the DANA Brief appears to meet this need. It can be used acutely in the remote and austere setting, and provides accurate assessment, including the assessment of cognitive constructs above and beyond those of other established measures (i.e., the MACE). Current findings provide at least preliminary evidence that the DANA Rapid may be a reasonable alternative to the more extended DANA Brief. Specifically, although the DANA Rapid excludes memory-based subtests, it includes the GNG and PRO, which appear to be more sensitive in the current analysis. It also includes two administrations of SRT, a measure of basic reaction time, which is one of the more sensitive measures of impairment after concussion.

Regarding our second hypothesis, since repeat testing over multiple days did not result in significantly improved DANA subtest performance, we did not find support for practice effects with repeated administration of the DANA Brief. It is unclear whether this was a function of the specific cognitive constructs assessed with the DANA Brief, as the extant neuropsychological literature suggests that practice effects do not respect any one domain or type of test (27). This is not a negative finding, as the goal of psychometric test development, used in serial monitoring, is to avoid practice effects (28).

The current findings have clear implications for the methodology or clinical practice of concussion assessment in multiple acute settings (e.g., emergency room, athletic field sideline). There is a need for future studies to assess the utility of the DANA Brief over longer injury and recovery intervals, as well as in concussed individuals. As discussed by McCrea, et al., the effect sizes of brief test instruments tend to diminish within the first week after injury (11). While this aligns with typical recovery curves in patients with uncomplicated mTBI, there is a subset that will continue to experience symptoms, including those in the cognitive domain. Therefore, showing sensitivity beyond these brief intervals, especially in concussed vs. non-concussed individuals, would further extend the utility of the DANA Brief. Future studies should counterbalance the administration of the DANA and the MACE to eliminate the potential of an order effect. In addition, there is certainly a need for generalizability, which may also be addressed in future studies by the use of a larger sample size. Studies have shown differential recovery curves, with interactions noted between age, gender, and symptom subset (29, 30). Including females, non-military, and age-varied participants would be worthwhile in this regard, as it would address the primary limitations of the current study. Although the cognitive assessment portion of the recently released MACE 2 is the same as is used in the MACE, the MACE 2 incorporates additional clinical assessment, including vestibular-ocular-motor screening. Therefore, more comprehensive future studies should compare the DANA and MACE 2. Finally, future studies would benefit from a larger sample size to increase power and reliability coefficients, bolstering the interpretation of diagnostic data.

While current findings provide at least preliminary evidence that the DANA Rapid version may serve as a clinically useful and improved neurocognitive concussion screen where professional medical evaluation is unavailable or delayed, future studies are needed to validate this potential by addressing identified study limitations. The use of the DANA Rapid in multiple acute settings would benefit service members, athletes and other individuals with concussion by minimizing the time between injury and diagnosis.

## Data Availability Statement

The datasets generated for this study are available on request to the corresponding author.

## Ethics Statement

This study was approved by the Institutional Review Board of the United States Army Medical and Materiel Command. Written informed consent was obtained from all participants in accordance with the Declaration of Helsinki.

## Author Contributions

JT conceived of the study. JT and AM designed the study. JT and SF were responsible for the acquisition of data. JP designed and reviewed the biostatistical analysis, which was conducted by JW. JP and BB interpreted the data. JP was the primary author responsible for the collaborative effort of drafting (JP, BB, EO'C, and AP) and revising (JP, BB, AM, SF, JW, and JT) the manuscript for intellectual content. All authors contributed to the article and approved the submitted version.

## Conflict of Interest

The authors declare that the research was conducted in the absence of any commercial or financial relationships that could be construed as a potential conflict of interest. The reviewer HS declared a shared affiliation with one of the authors AM, to the handling editor at time of review.
